# The Physicians’ Dose and Symptom Book

**Published:** 1871-12

**Authors:** 


*The Physicians' Dose and Symptoms Book.* By Joseph Wythes, M. D. Philadelphia: Lindsay & Blakiston, 1871.

This little work, which may be carried conveniently in the pocket, is intended to furnish assistance to the student of medicine, and to the general practitioner often saving him the trouble of referring to larger works. It contains an index of diseases and their proper treatment, some pharmaceutical preparations commonly used by the practitioner, a table of symptomology, and various other useful tables.

